# Assessing spatial variability and source identification of heavy metals in agricultural soils: A geostatistical and multivariate analysis of coastal eastern Zhejiang, China

**DOI:** 10.1371/journal.pone.0344184

**Published:** 2026-03-05

**Authors:** Jingwen Ji, Xiangyuan Wu

**Affiliations:** School of Public Administration, Zhejiang University, Hangzhou, Zhejiang, China; Ardakan University, IRAN, ISLAMIC REPUBLIC OF

## Abstract

Heavy metal pollution in coastal agricultural soils poses significant threats to food security, human health, and marine ecosystems. Effective prevention and control require systematic analysis of their spatial distribution and sources. This study integrated geostatistics, principal component analysis (PCA), positive matrix factorization (PMF), and finite mixture modeling (FMM) to comprehensively analyze the spatial variability and sources of five heavy metals (Cr, Pb, Cd, Hg, As) across 877 sampling sites in the coastal area of eastern Zhejiang. The results indicate that overall soil quality is good, though enrichment occurs at some sites due to anthropogenic activities. Pollution displays a spatial pattern of lower levels in the south and higher levels in the north. Pb is widely distributed, while Cd, Hg, and As are concentrated in agricultural plain areas. PMF-based source apportionment revealed that mobile sources (traffic) contributed the most (52.5%), followed by industrial sources (30.4%) and agricultural sources (17.1%). The consistency of multi-model results validated the reliability of source identification. By implementing precise management strategies based on pollution source contributions, it is expected to effectively curb the further deterioration of heavy metal pollution in agricultural soils in Zhejiang Province, gradually improve soil environmental quality, and ensure the safety of agricultural products and the sustainable development of agriculture.

## Introduction

In rapid industrialisation and urbanisation, agricultural soil pollution is becoming increasingly severe, significantly impacting human health and life [1]. Among the various forms of agricultural soil pollution, heavy metal contamination is of particular concern due to its widespread sources, high bioaccumulation, toxicity, and resistance to degradation [2]. It has been reported that approximately 10 million soil-contaminated plots exist globally, with over 50% (about 20 million hectares of land) affected by heavy metals [3,4]. According to the report “The state of soils in Europe”, published on December 19, 2024 by the Joint Research Center of the European Commission and the European Union Environment Agency, it can be seen that in the European Union (EU), about 19% of agricultural soils have at least one heavy metal that exceeds the limit values set by national legislation [5]. In the United States, 28% of soil samples contain heavy metal concentrations exceeding the Environmental Protection Agency’s (EPA) ecological soil screening levels [6]. In Russia, around 10% of the soil is deemed contaminated with “dangerous” or “moderately dangerous” heavy metals [7]. To effectively prevent and control soil heavy metal pollution, many countries have made significant efforts [8]. In the 1970s, the U.S. investigated heavy metal accumulation in its soils and crops, conducting risk assessments in contaminated areas [9]. In April 2024, the EU adopted the Soil Monitoring Act, marking the first soil-specific legislation in EU history, aiming to promote sustainable soil use and establish a legal framework to achieve soil health by 2050 [10]. In March 2022, China’s Ministry of Ecology and Environment issued the “Opinions on Further Strengthening Prevention and Control of Heavy Metal Pollution,” setting two primary objectives: first, to reduce emissions of key heavy metal pollutants from major industries by 5% compared to 2020 levels by 2025; and second, to comprehensively improve heavy metal pollution management, environmental risk prevention, and regulatory capacity by 2035 [11]. An overview of governance actions across countries highlights that investigating the spatial and source characteristics of heavy metals in agricultural soils is crucial for enhancing pollution monitoring and prevention [4,12–15].

Sources of heavy metal contamination in agricultural soils fall into two main categories: natural and anthropogenic [16]. Natural sources include the weathering of parent rocks and vegetation formation [17], while anthropogenic sources are more diverse, including industrial activities (e.g., industrial waste emissions, mining waste), urbanisation (e.g., automobile exhaust), agricultural activities (e.g., excessive pesticide use, irrigation wastewater), and military operations [18]. In recent years, source analysis of heavy metals in agricultural soils has gained prominence as a critical research topic, serving as a foundation for efficient pollution monitoring and land remediation [19]. Initially, researchers primarily employed Principal Component Analysis (PCA) and correlation analysis to qualitatively determine the sources of soil elements [20]. More recently, methods such as the Single pollution index method, Nemero pollution index, Ground Cumulative Index, and Enrichment Factor Method have been used to assess the potential risks of multiple heavy metals to plants, animals, and humans [21–24]. Additionally, techniques such as the Positive Matrix Factorisation (PMF) model and multivariate statistical analysis models are commonly used to qualitatively determine pollution sources and calculate the precise contribution of each source [25,26]. While these techniques provide valuable insights when used individually, they often yield only a fragmented understanding when applied in isolation. For instance, methods such as PCA and single pollution indices focus primarily on qualitative interpretation of pollution sources, whereas receptor models like PMF enable quantitative analysis of source contributions. It is therefore through the combined application of models with complementary emphases that a truly comprehensive and practical understanding of pollution sources can be achieved, thereby facilitating the development of precise and effective remediation strategies. In terms of sample selection, most studies on soil heavy metal pollution in China have focused on inland provinces, with less attention given to coastal areas. Furthermore, most studies concentrate on farmland around mining and industrial zones, with fewer studies examining farmland in river downstream areas or hilly regions [27–30]. To more comprehensively examine heavy metal contamination in agricultural soils, this study proposes selecting four cities in the eastern part of Zhejiang Province, China (Ningbo City, Taizhou City, Shaoxing City, and Jinhua City) as the sample area. The reasons for this selection are as follows: first, the Eastern Zhejiang is a key area in the Yangtze River estuary, situated within the River-Sea-Land Interaction Zone, and characterised by a mixed economic structure of agriculture, light industry, and trade, with rapid urbanisation [31,32]. Second, the Yangtze River Delta region, where Eastern Zhejiang is located, has been identified as a priority area for environmental protection due to its ecological sensitivity and high population density [33,34]. Soil contamination in this region directly affects food safety and public health for millions of residents [35]. Before this, agricultural development in Eastern Zhejiang was characterised by excessive use of chemical fertilisers, pesticides, and plastic films, leading to significant heavy metal pollution [35,36]. Third, the Eastern Zhejiang is also a common site for military exercises in the East China Sea, and as noted earlier, military operations can contribute to soil heavy metal pollution.

This study comprehensively applied geostatistical analysis, principal component analysis (PCA), positive matrix factorization (PMF), and finite mixture modeling (FMM) to investigate the spatial variability, pollution characteristics, and sources of heavy metals in agricultural soils of eastern Zhejiang. Based on 877 surface soil samples, the research aims to: (1) analyze the spatial distribution patterns of chromium (Cr), lead (Pb), cadmium (Cd), mercury (Hg), and arsenic (As); (2) perform source apportionment and quantify the contribution rates of heavy metal pollution in the study area; and (3) provide evidence-based targeted pollution control and sustainable soil management recommendations for coastal agricultural regions.

## Materials and methods

### Study area

Zhejiang Province (27°02′N–31°11′N, 118°01′E–123°10′E) is situated along China’s southeastern coast, forming the southern wing of the Yangtze River Delta. Bordered by the East China Sea to the east, Shanghai and Jiangsu to the north, Fujian to the south, and Jiangxi and Anhui to the west. The province spans approximately 365,500 km², comprising 105,500 km² of land and 260,000 km² of marine territory. Its topography descends terracedly from southwest to northeast: mountainous terrain dominates the southwest, hills the central region, and low-lying alluvial plains the northeast, encapsulated by the regional maxim “seven parts mountains, one part water, and two parts farmland (*QiShan Yi Shui Liang Fen Tian*)” [37]. Zhejiang experiences a subtropical monsoon climate with moderate humidity [38]. With per capita arable land at one-third of the national average, limited quantity, suboptimal quality, and scarce reserves, Zhejiang abandoned traditional agricultural models [39]. In 2003, guided by the “Lucid Waters and Lush Mountains Are Invaluable Assets *(Lvshui Qingshan Jiushi Jinshan Yinshan*)” philosophy, the province launched the “One Thousand Demonstration Villages and Rectification of Ten Thousand Villages (*Qiancun Shifan Wancun Zhengzhi*)” project to enhance rural ecosystems and livelihoods. In June 2019, Zhejiang province became China’s sole pilot province for modern eco-circular agriculture. In May 2021, the Central Government of the People’s Republic of China designated Zhejiang as a national common prosperity demonstration zone [40]. The province now achieves upper-middle-income economy status, with urban and rural disposable incomes consistently ranking first among Chinese provinces. Notably, all its prefectural cities exceed national average income levels.

Administratively, Zhejiang comprises 11 prefectural cities, geographically categorised as northern (Hangzhou, Jiaxing, Huzhou), southern (Wenzhou, Lishui, Quzhou), and eastern regions (Ningbo, Taizhou, Shaoxing, Jinhua, Zhoushan). This study focuses on four eastern cities: Ningbo (predominantly plains and low hills; 2024 GDP: 1,814.77 billion yuan), Jinhua (predominantly hills and basins; 2024 GDP: 692.55 billion yuan), Shaoxing (mixed plains, hills, and mountains; 2024 GDP: 836.9 billion yuan), and Taizhou (predominantly coastal plains and hills; 2024 GDP: 665.64 billion yuan). Zhoushan was excluded as 93.7% of its area comprises marine territory, offering limited relevance to the study.

### Soil sampling and sample determination

**Fig 1:**
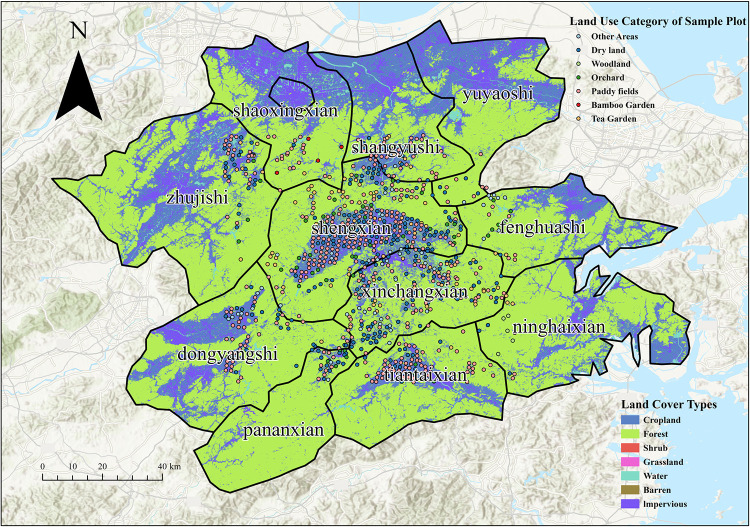
Distribution of sampling sites. (*Note:* Base map sources: Esri, HERE, Garmin, Intermap, Increment P Corp., GEBCO, USGS, FAO, NPS, NRCAN, GeoBase, IGN, Kadaster NL, Ordnance Survey, Esri Japan, METI, Esri China (Hong Kong), (c) OpenStreetMap contributors, and the GIS User Community [41]).

Sampling was conducted as part of the 2013 Zhejiang Province Agricultural Land Soil Heavy Metal Pollution Survey. Based on the distribution of basic farmland, topographic heterogeneity, and soil type variability, 877 agricultural soil sampling points were systematically established across the study area. Land use types at these points included paddy fields, upland fields, tea gardens, orchards, bamboo plantations, and forested areas. Paddy fields accounted for 48.35% of the total area, while upland fields constituted 27.94%. Soil types at sampling points encompassed red soil, paddy soil, coarse skeletal soil, purple soil, loess, and tidal soil, with red soil being the most prevalent at 51.31%. The spatial distribution of sampling points is illustrated in Fig 1. To more accurately reflect soil physicochemical properties, at least three samples were collected from each site, ultimately forming composite agricultural soil samples for each location. Sampling methods were selected based on field size and shape to ensure representativeness: spot sampling for small irregular fields, grid sampling for medium-sized regular fields, and serpentine sampling for large uniform fields. This approach followed standard soil sampling protocols to minimize spatial bias and enhance the representativeness accuracy of composite samples. For conventional crops, the sampling depth of the plow layer is 0–20 cm. For fruit trees and forest crops, the sampling depth is 0–60 cm. Differential GPS technology is used for precise location of sampling points. All soil samples were transported to the laboratory and air-dried indoors. After removing plant debris and stones, samples were ground and sieved through a 100-micron mesh. They were then sealed in amber glass bottles and stored at −20°C until analysis.

The indices for soil sample analysis included the total content of five heavy metal elements. Plasma optical emission spectrometry (ICP-OES) was used to determine Cr content, plasma mass spectrometry (ICP-MS) to determine Pb and Cd content, cold vapour-atomic fluorescence (CV-AFS) to determine Hg content, and hydride-atomic fluorescence (HG-AFS) to determine As content [42–45] The detection limits were 3.60 μg·g ⁻ ¹ for Cr, 0.91 μg·g ⁻ ¹ for Pb, 0.019 μg·g ⁻ ¹ for Cd, 0.53 ng·g ⁻ ¹ for Hg, and 0.069 μg·g ⁻ ¹ for As. Calibration curves were established with heavy metal standard solutions after every ten samples to ensure instrumental error remained within a 2% range. All reagents used in the analysis were of high purity. National standard reference materials, GSS-1 and GSS-4, were incorporated throughout the quality control process. All results were within the acceptable error tolerance range.

### Data analysis methods

#### Descriptive statistics and geostatistical analyses.

Descriptive statistical analyses of soil heavy metals were conducted using SPSS 22.0, including measures such as the arithmetic mean, extreme values, standard deviation, coefficient of variation, kurtosis, and skewness. Since the Ordinary Kriging Interpolation method in geostatistics requires normally distributed data. In the study, the data were tested for normality using the Kolmogorov-Smirnov (K-S) test in SPSS 22.0. Data that did not conform to a normal distribution were transformed using either a logarithmic or Box-Cox transformation. Then, we utilised the Ordinary Kriging interpolation model in the geostatistical analysis module in ArcGIS to determine the spatial variability of soil heavy metal elements.

#### Principal component analysis (PCA).

Principal Component Analysis (PCA) is a multivariate statistical method used for dimensionality reduction and feature extraction [46]. In this study, PCA is employed for exploratory analysis of heavy metal pollution data and preliminary determination of the number of pollution sources. First, the standardized heavy metal concentration data is processed through PCA. By calculating the eigenvalues and eigenvectors of the covariance matrix, the major components that explain the data variability are extracted.

The optimal number of pollution sources is determined based on the Kaiser criterion (retaining components with eigenvalues greater than 1), the cumulative variance contribution (reaching over 80%), and the elbow method from the scree plot. By analyzing the component loading matrix, the contribution of each heavy metal element to the different principal components is identified, allowing for a preliminary judgment of pollution source types and their characteristic element combinations*.*

#### Positive matrix factorization (PMF).

Positive Matrix Factorization (PMF) is a constrained non-negative matrix factorization method that can simultaneously provide quantitative estimates of both source profiles and source contributions [47]. This study uses the PMF model for precise identification and quantitative analysis of pollution sources. First, the heavy metal concentration data is log-transformed, and an uncertainty matrix is constructed, incorporating detection limits and analytical errors. Through an iterative factorization algorithm, the concentration matrix is decomposed into a source contribution matrix and a source profile matrix.

In the process of source identification, a multi-index decision system based on element enrichment characteristics and ratio relationships is established. For natural sources, the normalized concentrations of chromium and arsenic must both be greater than 0.15, with the chromium-arsenic ratio maintained within the typical range of 5−15. For industrial sources, at least two of the elements lead, cadmium, and mercury must have normalized concentrations greater than 0.1, and the lead-cadmium ratio should be between 50 and 200. For agricultural sources, a multi-index weighted scoring system is used, which comprehensively considers factors such as cadmium concentration, arsenic concentration, cadmium-arsenic co-enrichment characteristics, lead concentration range, mercury concentration levels, and the cadmium-lead ratio. The source is identified when the total score exceeds a threshold. For traffic sources, the normalized concentrations of lead and chromium must both be greater than 0.08, and the lead-chromium ratio should be maintained within the range of 0.3–0.8. Finally, the percentage contributions of each pollution source to the sample concentration are calculated through normalization, achieving a quantitative allocation of pollution sources.

#### The finite mixture model (FMM).

The Finite Mixture Model (FMM) is implemented using the Gaussian Mixture Model (GMM) for probabilistic clustering analysis of samples [48]. The principal component scores obtained from the previous PCA analysis are used as input features. The optimal number of clusters is determined through the Bayesian Information Criterion (BIC) and silhouette coefficient, with the Expectation-Maximization (EM) algorithm employed to estimate the parameters of each Gaussian distribution.

In terms of interpreting the clustering results, the heavy metal composition characteristics of each cluster center are compared with the source profiles obtained from Positive Matrix Factorization (PMF). A cosine similarity measure is used, and clusters with a similarity above 0.7 are identified as corresponding to specific pollution source types. This method provides each sample with the posterior probability of belonging to each cluster, enabling probabilistic source contribution assessment.

## Results

### Descriptive statistical analysis of heavy metal elements in agricultural soils

Descriptive statistics were performed on the concentrations of five heavy metals in 877 soil samples from the study area to obtain the minimum, maximum, mean, standard deviation, and coefficient of variation for each metal (see Table 1). The data revealed that the highest Cr concentration was 90.8 times greater than the lowest, with a mean value of 65.17 mg/kg. The highest Pb concentration was 77.7 times greater than the lowest, with a mean value of 35.08 mg/kg. The highest Cd concentration was 156 times greater than the lowest, with a mean value of 0.18 mg/kg. The highest Hg concentration was 291 times greater than the lowest, with a mean value of 0.10 mg/kg. The highest As concentration was 79.5 times greater than the lowest, with a mean value of 5.44 mg/kg. When compared with the background values of the soil environment in Zhejiang Province and national secondary standards, the average concentrations of the five heavy metals were lower than the environmental background values and the national standards. However, the highest concentrations exceeded these values. Specifically, the maximum concentrations of Cr, Pb, Cd, Hg, and As were 3.96, 11.73, 7.42, 12.65, and 7.29 times higher than the environmental background values for soil in Zhejiang Province, respectively. This suggests that the concentrations of these elements in the study area have significantly exceeded the natural levels and that soil quality at certain sample sites is compromised, likely due to industrial development and other factors.

**Table 1:** Agricultural soil heavy metal content (n = 877).

Element	Min	Max	Mean	SD	CoV	Background values of Zhejiang [50]	Grade II [51]
Cr	4.12	374.00	65.17	57.24	87.83	94.40	200.00
Pb	8.02	623.00	35.08	29.16	83.13	53.10	300.00
Cd	0.01	1.56	0.18	0.11	61.51	0.21	0.60
Hg	0.01	2.91	0.10	0.13	127.54	0.23	0.50
As	0.78	62.00	5.44	3.97	72.99	8.50	30.00

The units of heavy metals are mg.kg − 1; SD, standard deviation; CoV, coefficient of variable.

The coefficient of variation (CoV) indicates the degree of variability across sample points. A CoV of less than 20% represents low variability, 20%−50% indicates medium variability, 50%−100% denotes high variability, and over 100% reflects extreme variability [49]. The data showed that Hg exhibited extreme variability, while Cr, Pb, Cd, and As exhibited high variability. This indicates that the heavy metal concentrations in the study area are highly variable and discontinuous, likely due to the influence of external factors, suggesting that human activities are the primary source of pollution for these heavy metals.

### Spatial distribution of heavy metal contamination in agricultural soils

#### Spatial variation characteristic functions.

The nugget effect is a key indicator in geostatistics for measuring the randomness of spatial variability. A higher value suggests that the spatial distribution of heavy metals is predominantly influenced by localized, stochastic anthropogenic activities such as industrial emissions, transportation sources, or agricultural fertilization. Conversely, a lower value reflects the dominant role of structural factors like natural soil-forming processes. By comparing the parameters of each fitting test, the best-fitting models for the five heavy metal elements and their associated parameters were determined (see Table 2). These parameters reflect the variability characteristics of heavy metal content in soil. By comparing and analysing these parameters, it is possible to theoretically understand the spatial distribution characteristics of soil heavy metals [50,52,53].

**Table 2:** Semivariogram theoretical models and parameters of agricultural soil heavy metal content.

Element	Theoretical model	Nugget (Co)	Sill (C1 + C0)	Range (R)	Co/(Co + C1)
Cr	Exponentia	0.122879	0.570438	0.191659	21.54
Pb	Gaussian	0.088583	0.060167	0.098436	147.23
Cd	Spherical	0.229899	0.066878	0.182511	343.76
Hg	Gaussian	0.335466	0.056452	0.076036	594.25
As	Gaussian	0.094642	0.073730	0.019007	128.36

The nugget value (C0) represents a type of variation not caused by the spacing of sampling points. It is random and reflects spatial variation influenced by random factors (e.g., socio-economic factors). The abutment value (C1 + C0) refers to the extreme value of the semi-variance observed at different sampling spacings. It reflects spatial variation caused by natural factors (e.g., soil-forming parent material, topography) and socio-economic factors (e.g., fertiliser application, cropping systems). This value includes both random and structural variability. The ratio of the nugget value (C0) to the abutment value (C1 + C0) is an important indicator of the degree of spatial variability in regional variables, commonly referred to as the nugget effect. This ratio helps to determine whether natural (structural) or anthropogenic (stochastic) factors primarily influence spatial variation. When C0/(C1 + C0) < 25%, it suggests that spatial variation is dominated by structural factors, indicating a strong spatial correlation primarily controlled by natural factors with minimal human influence. When 25% ≤ C0/(C1 + C0) ≤ 75%, it indicates moderate spatial correlation. When C0/(C1 + C0) > 75%, it indicates that spatial variation is primarily random, resulting in weak spatial correlation influenced more by human factors. The data reveal the following nugget effects for the five heavy metals, from smallest to largest: Cr (21.54%), As (128.36%), Pb (147.23%), Cd (343.76%), and Hg (594.25%). Only Cr has a nugget effect of less than 25%, while the nugget effects of the remaining four metals exceed 75%.

The maximum correlation distance, or range, refers to the distance at which the variability function reaches the abutment value, indicating the spatial autocorrelation range of the elements. Changes in this range reflect shifts in the primary variability process of the soil elements. A larger variance range suggests stronger homogeneity of the element in the soil, while a smaller range indicates weaker homogeneity, with more pronounced local variations and a more complex overall distribution. As shown in Table 2, the ranges for the five heavy metals—Cr, Pb, Cd, Hg, and As—are all small. This suggests that the distribution of these metals in soil within a narrow range of variation should not be overlooked.

#### Spatial distribution characteristics of heavy metal pollution in agricultural soil.

**Fig 2:**
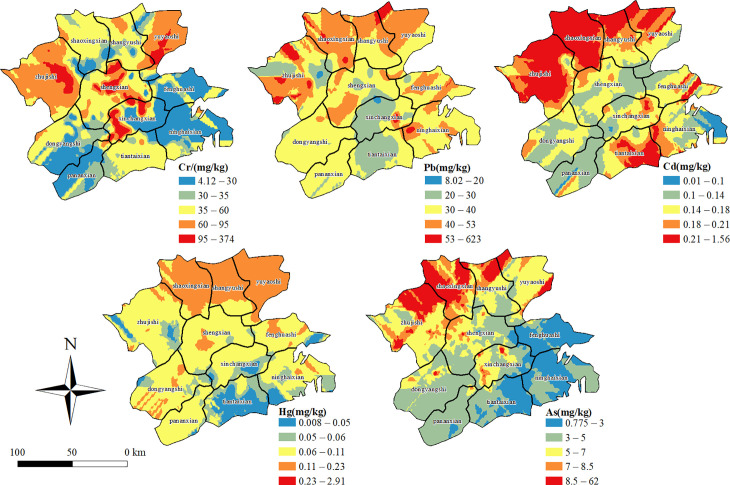
Spatial distribution of heavy metal concentrations in study area soils. (The map was created using ArcGIS software. The underlying administrative boundary data are from Natural Earth (https://www.naturalearthdata.com/), which is in the public domain).

Using the geostatistical analysis function of the extended ArcGIS module (Geostatistical Analyst), the Ordinary Kriging interpolation method was applied to establish the spatial variability pattern of heavy metal elements in the soil (Fig 2). In the figure, the heavy metal content is divided into ten levels.

As shown in Fig 2, the distribution of soil heavy metal content in the study area shows the characteristics of low in the south – high in the north.The high value areas of Cr element appear in the territory of Shengzhou City and Xinchang County, which, as one of the top 100 industrial counties (cities) in the country in 2018, can be seen that there are individual chemical enterprises in the region that produce more serious point-source pollution.The high value areas of Pb element have a wider distribution, covering almost the entire eastern part of Zhejiang Province. Considering that the main reason for the influence of Pb is automobile exhaust, which is related to the local population density and the number of fuel vehicles, the government should introduce policies to encourage the public to use public transportation and new energy vehicles to travel.The high value areas of Cd are found in Yuyao, Shangyu, Shaoxing and Tiantai counties, the high value areas of Hg are found in Yuyao, Shangyu and Shaoxing counties, the high value areas of As are found in Yuyao, Shangyu and Shaoxing counties, and the high value areas of Hg are found in Yuyao, Shangyu and Shaoxing counties. in Yuyao City, Shangyu City, Shaoxing County and Zhuji City. These counties (cities) are all located in the Ningshao Plain, which is one of the important grain, cotton, hemp and freshwater fish production areas in Zhejiang Province. It can be seen that the application of chemical fertilizers and pesticides is still high in agricultural activities in the Ningshao Plain, which needs further greening and ecological transformation.

### Tracing heavy metal pollution in the soil

#### Preliminary identification of pollution sources based on PCA.

The correlation between the content of the five heavy metals was analysed using the Pearson coefficient method, as shown in Table 3. It is evident that there is a significant (P < 0.05) or highly significant positive correlation (P < 0.01) between Pb-Cd, Pb-Hg, Cd-Hg, and Hg-As, indicating that Pb, Cd, Hg, and As are homologous.

**Table 3:** Correlation coefficients of agricultural soil content.

	P_Cr	P_Pb	P_Cd	P_Hg	P_As
P_Cr	1.000				
P_Pb	−.163**	1.000			
P_Cd	−.028	.379**	1.000		
P_Hg	−.074*	.155**	.093**	1.000	
P_As	.027	.052	.033	.076*	1.000

Note that * and ** denote statistically significant correlation at the 0.05 and 0.01 probability levels, respectively.

**Fig 3:**
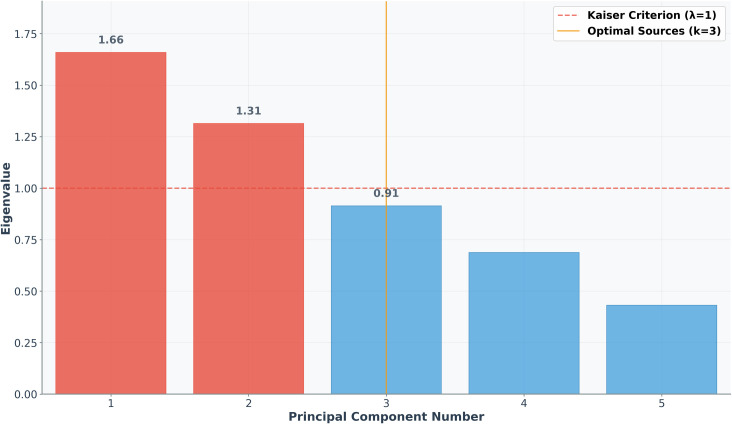
Scree plot of principal component eigenvalues.

PCA, as a classical multivariate statistical dimensionality reduction technique, effectively identifies correlation patterns among heavy metal elements and provides a crucial basis for the preliminary identification of pollution sources. The results shown in Fig 3 reveal that the eigenvalues of the first three principal components are 1.66, 1.31, and 0.91, respectively. The first two components have eigenvalues greater than 1, satisfying the Kaiser criterion [54]. Although the eigenvalue of the third component is slightly below the Kaiser criterion threshold, it still holds some explanatory significance. Considering the cumulative variance contribution and the actual complexity of pollution sources, three principal components were selected for subsequent analysis, with a cumulative variance contribution rate of 77.7%.

**Fig 4:**
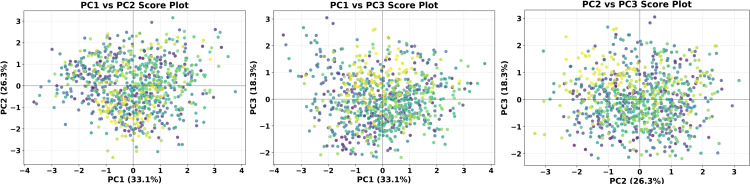
Principal component score scatter plots.

Fig 4 further illustrates the distribution characteristics of the samples in the reduced-dimensional space. The PC1-PC2 score plot shows a relatively concentrated distribution of the samples, indicating that the soil heavy metal pollution in the study area exhibits a certain homogeneity. The PC1-PC3 and PC2-PC3 score plots demonstrate the spatial differentiation patterns of the samples under different combinations of principal components, providing spatial references for identifying the influence range of different pollution sources.

**Fig 5:**
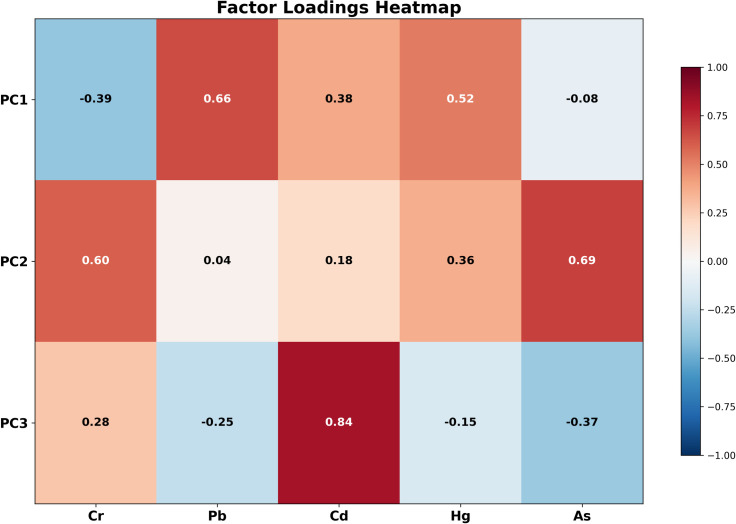
Factor loadings heatmap.

Fig 5 shows the results of the principal component analysis. The PC1 explains 33.1% of the total variance, with Pb (0.66) and Hg (0.52) showing high positive loadings, while Cr (−0.39) exhibits a negative loading. The high loading of Pb and Hg suggests that this component mainly reflects industrial pollution sources, particularly those arising from industrial processes such as non-ferrous metal smelting, coal-fired power generation, and chemical production.

The PC2 explains 26.3% of the total variance, with Cr (0.60) and As (0.69) displaying significant positive loadings. The co-enrichment pattern of Cr and As primarily reflects mobile source pollution, with Cr mainly originating from vehicle exhaust emissions and road wear, while As is associated with gasoline combustion and tire wear.

The PC3 explains 18.3% of the total variance, with Cd (0.84) exhibiting a very high loading, while As (−0.37) shows a negative loading. The high loading of Cd suggests its primary source is agricultural activities, particularly the use of phosphate fertilizers, pesticides, and livestock farming.

In summary, the PCA analysis preliminarily identifies three major pollution sources in the study area: industrial pollution, mobile source pollution, and agricultural pollution. This result lays a significant foundation for subsequent precise source apportionment analysis.

#### Pollution source apportionment and quantification based on PMF.

**Fig 6:**
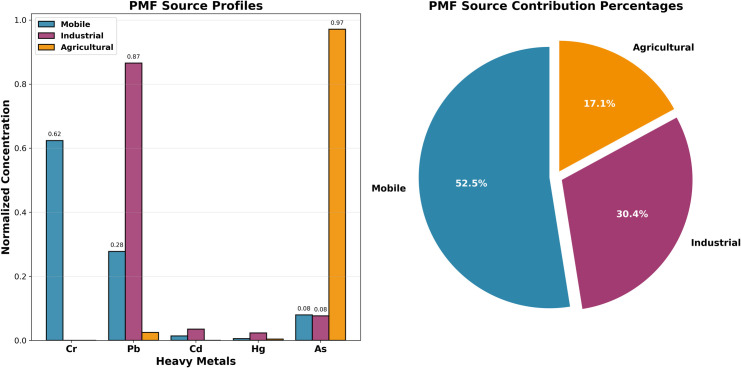
Source profiles and contribution percentages of PMF analysis.

PMF, as an advanced receptor model, can quantitatively identify pollution sources and estimate the contribution of each source to pollutant concentrations at receptor sites. Based on the three pollution sources determined by the preliminary PCA analysis, this study employed the PMF model to conduct precise source apportionment and quantitative analysis of soil heavy metal pollution in the study area. As shown in Fig 6, the source apportionment results of the PMF model were highly consistent with the preliminary identification of pollution sources from the PCA analysis. The model successfully resolved three pollution sources with clear physical significance. Among them, the mobile source was characterized primarily by Cr, with a standardized concentration of 0.62, indicating that the mobile source is the main contributor to Cr pollution in the soil. Mobile source pollution primarily originates from traffic-related activities such as vehicle exhaust emissions, tire wear, brake wear, and road dust. The enrichment of Cr in the mobile source reflects the widespread use of automotive catalytic converters, engine component wear, and chromium-containing alloy materials [55,56].

The industrial source exhibited extremely high enrichment of Pb, with a standardized concentration as high as 0.87, while Hg also showed a certain contribution (standardized concentration of 0.05). This combination of characteristics typically reflects the composite pollution features of industrial activities, mainly including non-ferrous metal smelting, coal-fired power generation, chemical production, and other high-temperature industrial processes. The high loading of Pb indicates that the industrial source is the dominant factor for soil Pb pollution, while the presence of Hg further confirms the contribution of high-temperature industrial processes such as coal combustion [57,58].

The agricultural source was absolutely dominated by As, with a standardized concentration of 0.97, showing a typical single-element dominance characteristic. The extreme enrichment of as in the agricultural source is mainly related to the application of phosphate fertilizers, as phosphate ores naturally contain high concentrations of arsenic. Additionally, historically used arsenic-containing pesticides and the application of livestock manure may also contribute to soil arsenic pollution [59,60].

Meanwhile, the quantitative analysis results of the PMF model indicated significant differences in the relative contributions of the three pollution sources to soil heavy metal pollution in the study area. The mobile source contributed the most, accounting for 52.5% of the total pollution load, making it the primary source of soil heavy metal pollution in the study area. This result reflects the significant impact of highly developed transportation and continuously growing vehicle ownership on soil environmental quality in Zhejiang Province as an economically developed region. The high contribution rate of mobile source pollution suggests that soil environmental supervision should focus on traffic-intensive areas.

The industrial source contributed the second highest proportion, accounting for 30.4% of the total pollution load, reflecting the important impact of industrial activities on soil heavy metal pollution. As a major manufacturing province with densely distributed industrial enterprises, especially the development of heavy chemical and metal processing industries, Zhejiang Province has exerted a continuous cumulative impact on the soil environment.

The agricultural source contributed relatively less, accounting for 17.1% of the total pollution load, mainly reflecting the contribution of fertilizer and pesticide application in agricultural production activities to the accumulation of heavy metals in soil. Although the proportion is relatively low, given that the study object is agricultural land soil, pollution from agricultural sources still requires sufficient attention.

#### Probabilistic clustering of pollution sources based on PCA-FMM.

**Fig 7:**
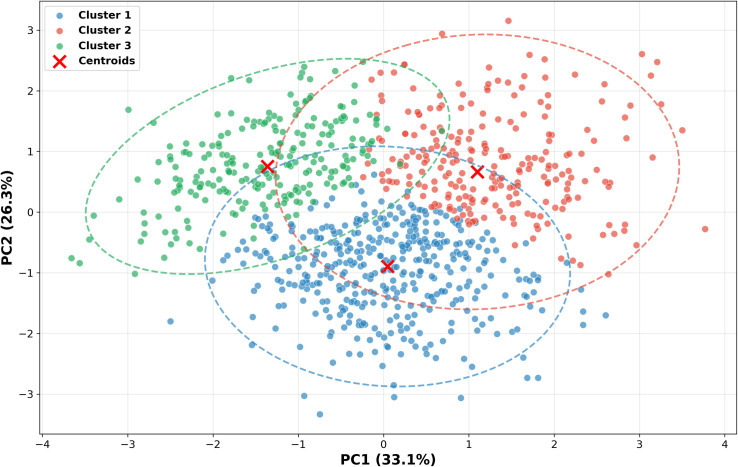
Probabilistic clustering of pollution sources based on PCA-FMM.

Building upon the prior PCA dimensionality reduction results, this study employs the FMM method to conduct probabilistic clustering analysis in the principal component space, aiming to reveal the spatial distribution patterns of soil samples under the influence of different pollution sources. As shown in Fig 7, the FMM probabilistic clustering analysis successfully identified three statistically significant clusters in the PC1–PC2 principal component space. The probability ellipse boundaries of each cluster are distinct, indicating clear statistical demarcations among regions influenced by different pollution sources, thereby providing a quantitative basis for spatial management of pollution sources.

Cluster 1 is primarily distributed in the middle-lower region of the PC1–PC2 space, with a centroid position of approximately (–0.5, –0.8). This cluster contains the largest number of samples and exhibits a relatively concentrated elliptical distribution pattern. Based on the loadings characteristics of PC1 and PC2, this cluster corresponds to regions dominated by mobile source pollution, mainly reflecting the impact of transportation activities on heavy metal contamination in soil.

Cluster 2 is distributed in the upper-right region of the PC1–PC2 space, with a centroid position of approximately (1.8, 0.6), displaying relatively dispersed distribution characteristics. This cluster corresponds to regions dominated by industrial source pollution, reflecting the spatially heterogeneous influence of industrial activities. These areas are mainly concentrated around industrial parks, smelting enterprises, and historically contaminated industrial sites, with their spatial dispersion reflecting the characteristics of industrial point source pollution.

Cluster 3 is primarily distributed in the left region of the PC1–PC2 space, with a centroid position of approximately (–2.2, 1.0), exhibiting a moderate degree of spatial aggregation. This cluster corresponds to regions dominated by agricultural source pollution, mainly reflecting the contribution of agricultural activities to heavy metal contamination in soil. These areas typically include intensive agricultural production zones, facility agricultural areas, and farmlands with long-term phosphate fertilizer application.

Crucially, the FMM clustering results show strong correspondence with the PMF source apportionment results: the distribution patterns of the three clusters in the principal component space highly align with the three types of pollution sources identified by PMF, validating the spatial rationality of the pollution source apportionment results. Cluster 1 has the largest number of samples, corresponding to the highest contribution rate (52.5%) from mobile sources in the PMF results, while the sample distribution proportions of Cluster 2 and Cluster 3 are also consistent with the contribution rates of industrial sources (30.4%) and agricultural sources (17.1%), respectively. The clear probability ellipse boundaries of each cluster indicate well-defined statistical limits for regions influenced by different pollution sources.

## Discussions

### Comparison and validation of multi-model results

**Fig 8:**
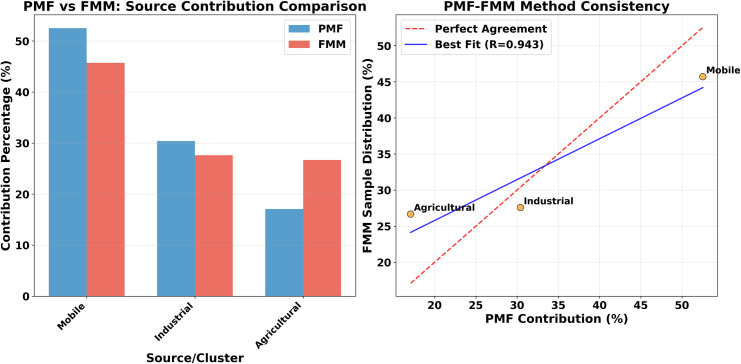
Comparison and validation of PMF and FMM model results.

As shown in Fig 8, the PMF and FMM methods demonstrate strong consistency in quantifying the contributions of the three pollution source categories, though some methodological differences exist. The contributions of mobile and industrial sources show minor differences, with PMF results slightly higher than those of FMM. In contrast, the agricultural source contribution exhibits the largest discrepancy, with PMF estimating 17.1% and FMM estimating 27.0%, a difference of 9.9 percentage points.

These differences can be attributed to the distinct algorithmic principles of the two methods. PMF is highly sensitive to concentration variations of source-specific elements, enabling precise capture of subtle changes in source contributions. In comparison, FMM focuses more on the distribution patterns of samples in multidimensional space and demonstrates stronger robustness to outliers and noise. PMF, based on factorization principles, directly decomposes source contributions by minimizing an objective function, while FMM, grounded in probabilistic statistics, identifies sample cluster distributions using Gaussian mixture models. The differing mathematical foundations of the two algorithms lead to variations when addressing complex pollution source mixtures [61].

Overall, the pollution source identification results obtained from these two independent methods are highly consistent, with a correlation coefficient of 0.943, validating the reliability of the source apportionment results. The quantitative precision of PMF and the spatial distribution analysis of FMM complement each other, providing a more comprehensive and robust scientific basis for pollution source analysis. The consistency observed despite differing algorithmic principles indicates that the identified three pollution source categories possess clear physical meaning and statistical significance.

### Implications for soil pollution remediation

Based on the results of pollution source apportionment using a multi-method fusion approach combining PCA-PMF-FMM, this study provides a scientific theoretical basis and practical guidance for the remediation of heavy metal pollution in agricultural soils in Zhejiang Province. The contribution patterns revealed by PMF pollution source apportionment—mobile sources (52.5%), industrial sources (30.4%), and agricultural sources (17.1%)—offer important insights for developing differentiated and targeted pollution prevention and control strategies.

For mobile sources: Implement traffic emission reduction measures in identified high-lead contamination areas (particularly the northern region with elevated pollution levels), including: (a) promoting electric vehicle infrastructure in agricultural zones; (b) establishing green buffer zones along major highways traversing farmland; (c) conducting regular monitoring of roadside soils.

For industrial pollution sources: Strengthen oversight of industrial emissions in key pollution zones (Shenzhou and Xinchang counties) through: (a) strict enforcement of wastewater and exhaust emission standards for metallurgical and chemical enterprises; (b) mandatory soil remediation plans for historically polluted industrial zones; (c) establishment of real-time heavy metal emission monitoring systems in industrial parks.

For agricultural pollution sources: Promote sustainable agricultural practices in the Ning-Shao Plain region, specifically including: (a) Reducing phosphorus fertilizer application through precision agriculture technologies; (b) Implementing organic agriculture certification programs with heavy metal testing requirements; (c) Developing arsenic-safe agricultural guidelines for affected areas. By implementing targeted remediation strategies based on pollution source contributions, it is anticipated that further deterioration of heavy metal contamination in agricultural soils across Zhejiang Province can be effectively controlled. This approach will progressively improve soil environmental quality, safeguard the quality and safety of agricultural products, and ensure sustainable agricultural development.

## Conclusions

This study employed a combination of analytical methods, including geostatistics, PCA, PMF, and FMM, to systematically investigate the spatial distribution patterns, pollution characteristics, and sources of heavy metals (Cr, Pb, Cd, Hg, As) in agricultural soils in the coastal region of eastern Zhejiang. The results indicate that heavy metal pollution in soils is predominantly anthropogenic, with significant spatial heterogeneity and generally higher pollution levels in the northern part of the study area compared to the south. Source apportionment using the PMF model revealed that mobile sources, industrial sources, and agricultural sources were the primary contributors, with respective contribution rates of 52.5%, 30.4%, and 17.1%. The FMM clustering results further confirmed the spatial differentiation among these pollution sources.

However, this study has certain limitations. First, the source apportionment relied on receptor models and statistical methods without validation against specific industrial enterprise locations and emission inventories, which constrains the precise quantification of source contributions. Second, the analysis was based solely on surface soil samples and did not capture the behavior of heavy metals in terms of vertical migration and bioavailability. Furthermore, the influence of temporal variations on pollution accumulation was not considered, making it difficult to dynamically assess pollution trends.

Future research should focus on the following aspects: First, integrating high-precision industrial emission data with spatial information technologies to establish quantitative source–receptor relationships. Second, conducting studies on heavy metal speciation and vertical distribution to enhance understanding of their environmental behavior and ecological risks. Third, establishing long-term monitoring networks to elucidate spatiotemporal evolution patterns of pollution, thereby providing a scientific basis for dynamic regulation and targeted management. Building on these efforts, a soil pollution prevention and control system based on major source identification and zonal management can be further developed to support regional agricultural sustainable development and ecological security maintenance.
